# Emotion regulation and its relation to symptoms of anxiety and depression in children aged 8–12 years: does parental gender play a differentiating role?

**DOI:** 10.1186/s40359-018-0255-y

**Published:** 2018-08-20

**Authors:** M. E. S. Loevaas, A. M. Sund, J. Patras, K. Martinsen, O. Hjemdal, S.-P. Neumer, S. Holen, T. Reinfjell

**Affiliations:** 1 Department of Psychology, NTNU, Norwegian University of Science and Technology, Trondheim, Norway; 2 Department of Child and Adolescent Psychiatry, St. Olavs University Hospital, Trondheim, Norway; 3 Centre for Child and Adolescent Mental Health, RBUP East and South, Oslo, Norway; 4 Regional Center for Child and Youth Mental Health and Child Welfare, NTNU Norwegian University of Science and Technology, Trondheim, Norway; 5 RKBU – North, Health Sciences Faculty, UiT The Arctic University of Norway, Tromso, Norway

**Keywords:** Emotion regulation, Anxiety, Depression, Children

## Abstract

**Background:**

Symptoms of anxiety and depression are prevalent and highly comorbid in children, contributing to considerable impairment even at a subclinical level. Difficulties with emotion regulation are potentially related to both anxious and depressive symptoms. Research looking at maternal contributions to children’s mental health dominates the literature but ignores the potentially important contributions of fathers.

**Method:**

The present study is part of the Coping Kids study in Norway, a randomized controlled study of a new indicated preventive intervention for children, EMOTION. EMOTION aims to reduce levels of anxious and depressive symptoms in children aged 8–12 years. Using cross sectional data and multiple regression analyses, we investigated the relations between anxious and depressive symptoms and emotion regulation in *n* = 602 children. Symptoms were reported by the child, mothers and fathers. Emotion regulation was reported by mothers and fathers.

**Results:**

Symptoms of anxiety, as reported by parents, were associated with poorer emotion regulation. This association was also demonstrated for depressive symptoms as reported by both parents and children. When analyzing same gender reports, parental gender did not differentiate the relationship between anxiety symptoms and emotion regulation. For depressive symptoms, we did find a differentiating effect of parental gender, as the association with dysregulation of emotion was stronger in paternal reports, and the association with adaptive emotion regulation was stronger in maternal reports. When using reports from the opposite parent, the emotion regulation difficulties were still associated with depressive and anxiety symptoms, however exhibiting somewhat different emotional regulation profiles.

**Conclusion:**

Problems with emotion regulation probably coexists with elevated levels of internalizing symptoms in children. In future research, both caregivers should be included.

**Trial registration:**

The regional ethics committee (REC) of Norway approved the study. Registration number: 2013/1909; Project title: Coping Kids: a randomized controlled study of a new indicated preventive intervention for children with symptoms of anxiety and depression. ClinicalTrials.gov; Protocol ID 228846/H10.

## Background

### Emotion regulation, anxiety and depression

The regulation of emotions is important in children’s adaptive development, playing a role in, for example, executive cognitive functions and social competence [1, 2], as well as in the development of psychopathology [3]. Anxiety and depressive disorders in children are global health concerns, with an estimated three-month prevalence of 2.2% for depression and 2.4% for anxiety [4]. Comorbidity rates between anxiety and depression are as high as 30% [4, 5]. In addition, symptoms of anxiety and depression that do not meet diagnostic criteria contribute to considerable impairment [5, 6], and subclinical symptoms might develop into disorders [7, 8]. Preventive interventions for anxiety and depression are important in reducing the development of disorders later in life, and emotion regulation is one potentially relevant factor to consider [3].

Emotion regulation is defined as “the extrinsic and intrinsic processes responsible for monitoring, evaluating, and modifying emotional reactions, especially their intensive and temporal features, to accomplish one’s goal” [9]. The success of emotion regulation depends on the adaptation of responses to situational demands [10], and while this ability develops throughout life, children have acquired their primary regulation strategies by approximately the age of seven [11]. The strategies used to regulate emotions are diverse and include, for example, help seeking, avoidance, attentional redirection, suppression, and problem solving. Development of these strategies is complex and interacts with genetics, biology, cognition, temperament, social environment, and learning [11].

Theoretically, children who repeatedly fail to regulate their emotions in accordance with the context are at greater risk of developing internalizing symptoms. Barlow and colleges [12] introduced a triple vulnerability model for internalizing symptoms, consisting of biological and psychological vulnerabilities combined with negative early learning situations. When children perceive a situation as uncontrollable and/or a strong unwanted feeling occurs, this leads the individual to initiate emotion regulation efforts. If emotion regulation is ineffective, this leads to an increase in the unwanted feelings, which may again lead the individual into a negative cycle with increasing psychological distress and poor attempts at emotion regulation. Over time, this might develop into an anxiety or depressive disorder [12]. Others have developed similar theories for specific disorders such as depression [13] and anxiety [14], where repeatedly failing to downregulate unwanted feelings leads to an increased risk of disorders.

In support of these theories, one longitudinal study found that poor emotion regulation skills predicted internalizing symptoms in children [15]. This result is in line with a cross-section study by Zeman and colleges [16] indicating associations between internalizing symptoms and poor emotion regulation. Additionally, children diagnosed with an anxiety disorder reported more dysregulation of affect compared to a control group of non-anxious children [17]. The use of less effective emotion regulation strategies has also been associated with depression for both children and adolescents [18, 19]. Longitudinal findings indicate that difficulties with emotion regulation in pre-adolescence could also be a risk-factor for both depression and anxiety [20, 21].

Depressive symptoms are mainly linked to dysregulation of dysphoria and sadness [13] and anxious symptoms to dysregulation of fear [14]. Symptoms are fluctuating phenomena, with varying prevalence among individuals [4]. In contrast, emotion regulation is a more stable trait [3, 11] that includes the regulation of all possible emotions using a broad range of regulative strategies [11]. Theoretically, internalizing symptoms and emotion regulation are related but distinct phenomena.

The association between youth psychopathology symptoms and emotion regulation was confirmed in a recent meta-analytic study [3]. However, a large portion of the studies included in the review used an American sample and focused on adolescents. Culture potentially influences the association between internalizing symptoms and emotion regulation [22]. Replication in other cultures is therefore important to broaden our understanding of how internalizing symptoms and emotion regulation are associated.

### Parental differences

Informant difference between child and parent is common, and in studies on anxious and depressive symptoms moderate discrepancies are typically reported [23]. Parental reports of children’s internalizing symptoms are considered valid [24]. Informant differences have traditionally been viewed as measurement error, but resent research have pointed to this instead being a reflection of different perspectives and relationships, and providing clinically meaningful information [25].

Studies of how parents report children’s symptoms have mainly found small differences, with mothers generally reporting more problems than fathers [26, 27]. Mothers rate their children higher on social-emotional competence and dysregulation problems than do fathers [27]. Parental agreement is higher for externalizing than for internalizing difficulties, and parental agreement has been found to be moderated by children’s age, gender and socioeconomic status [26]. Consequently, one would expect parents’ reports of their children’s emotion regulation capacities to differ. Multiple informants are generally viewed as a strength in research [26], but including both parents as informants may be costly and time-consuming. Is it necessary to include both parents in research regarding emotion regulation? In order to answer this question, we must compare maternal and paternal reports of child emotion regulation.

Parents are actively involved in the external regulation of the child’s emotions as well as in the process of teaching the child internal regulation [11]. As a result, one could expect children’s expressed emotion regulation to differ between situations with different caregivers. In addition, mothers and fathers might make divergent interpretations of a child’s behavior in terms of emotion regulation. Differences between parental reports of children’s symptoms may therefore reflect actual differences in the relation between children and parents [25], and in this context, may reflect actual differences in the child’s emotion regulation ability in relation to the different caregivers. A better understanding of informant differences might therefore contribute to a better understanding of the child’s emotion regulation capacities. Research focusing only on mothers ignores the potential differentiating paternal role. This uncertainty underlines the importance of including both caregivers in research.

### Control variables

There seems to be an association between experiencing stressors and poor emotion regulation, contributing to the increased risk of internalizing symptoms [28, 29]. Similarly, parental mental health problems are risk factors for childhood psychopathology, and parental mental health influences children’s development of emotion regulation [30]. Sociodemographic factors (SES), such as parental education and the family economy, also influence children’s mental health [31] and possibly the association between internalizing symptoms and emotion regulation [32]. Based on this, it is important to control for the influence of sociodemographic factors, parental mental health and experienced stress to understand the relationship between symptoms of anxiety and depression and emotion regulation.

In addition, we controlled for the child’s age and gender, both of which are important demographic factors in the development of anxiety and depression [4, 29]. Emotional regulation continues to develop in middle childhood, and there may be differences related to age [11]. There are also potential gender differences in emotion regulation [17].

This article examines the associations between anxious and depressive symptoms and difficulties in emotion regulation in Norwegian school children aged 8–12 years. Both mothers and fathers reported on their child’s emotion regulation capacities, and we further investigated whether parental gender has a differentiating role. To our knowledge, these questions have not previously been investigated in a Norwegian child population with emotional problems, and very few relevant studies have been conducted worldwide.

We hypothesize that symptoms of anxiety and depression as reported by the child, mother and father will be negatively associated with emotion regulation skills as reported by mothers and fathers when controlling for the child’s age and gender, family economy, parental education, parental mental health, and chronic and acute stressors. We further examined whether the association between internalizing symptoms and emotion regulation differed depending on the informant being mother or father.

## Method

### Procedure

The present study uses baseline data from the Coping Kids study in Norwegian schools. Coping Kids is a national cluster randomized controlled study of an indicated group-based cognitive behavioral therapy (CBT) intervention, EMOTION, for children between the ages of 8 and 12 with elevated anxiety and depressive symptoms. Participants came from three sites across Norway, including both urban and rural areas. Schools volunteered to participate in the project, and children in grades 3, 4, 5 and 6 (corresponding to age range of 8–12 years) received written invitations to participate in the screening. Taking part in the screening required written informed consent from a parent and expressed interest from the child. Children answered questionnaires electronically at school, and parents did so at home via e-mailed links. Data used in the present study are cross-sectional baseline data, collected between autumn 2014 and spring 2016; new children entered the study every semester. For a complete description of the study and protocol, see Patras and colleague [33].

### Participants

A total of 1686 children were screened for symptoms of anxiety and depression, and 873 children were invited to participate in an intervention study based on scoring one SD or above a population mean on measures of symptoms of anxiety and/or depression. Seven children were excluded due to exclusion criteria (mental retardation, autism, or severe behavioral disturbance), and 71 were randomly excluded due to lack of resources (lack of group leaders). Parents of the included children (*n* = 795) were invited to participate in the study, and the parental response was 78.5%. For the present study, inclusion required the availability of parental data; 624 children had at least one parent participate in the study. A total of 850 parents (*n* = 299 fathers, and *n* = 550 mothers) were included in the present study, of these, 226 children had both parents participate in the study.

There were no significant differences between children with and without parental response regarding age or symptom levels of anxiety and depression. Sociodemographic variables, stress experienced by the child, and parental mental health were only reported by parents. Therefore, no comparisons between children with and without parental data were computable for these variables.

### Sociodemographics

In our sample, 94.7% of the children, 88.9% of the mothers, and 88.8% of the fathers reported Norway as place of birth. The mean age of the children was 10.1 (*SD* = 0.90) years. Girls represented 58.1% of the sample, and this gender difference was significant (*t* = 80.15, *p* < 0.001). As symptoms of depression, and potentially of anxiety, are more prevalent in girls in the current age group [4, 29], this gender difference is considered representative for this population.

Parents rated the economic situation of the family on a five-point scale ranging from one *(less than 350.000 NOK)* to five (*over 1 million NOK*). A total of 81.2% rated their family income above 500.000 NOK, which is equivalent to the median income in Norway [34].

Parents rated their education levels individually from one *(= ten years of primary school*) to five (*= four years or more of college/university*). A total of 30.2% of fathers and 60.4% of mothers reported four or more years of college/university, compared to 32.2% for the general population in Norway (35.6% of females and 28.7% of males) [35].

### Measures

#### Mood and feeling questionnaire – Short form (SMFQ)

The 13-item SMFQ child and parental versions were used to screen depressive symptoms experienced over the previous 2 weeks [36]. Higher scores indicate higher levels of depressive symptoms. In the present sample, Cronbach’s alpha was good for both parental reports (mothers α = 0.88, and fathers α = 0.88) and child self-reports (α = 0.81). Norwegian norms for the SMFQ are available [37].

#### Multidimensional anxiety scale for children (MASC)

The 39**-**item MASC child and parental version was used to screen anxious symptoms experienced over the previous 2 weeks [38]. Higher scores indicate higher levels of anxiety symptoms. In the present sample, Cronbach’s alpha was excellent for both parental reports (mothers α = 0.90, and fathers α = 0.90) and good for child self-reports (α = 0.85). The MASC is validated in Norway [39] as well as internationally [38].

#### Emotion regulation checklist (ERC)

The 24-item ERC [40] is a questionnaire assessing children’s emotion regulation as reported by parents, validated by Shields and Cicchetti [40]. The questionnaire was previously validated in European samples [41] in addition to the original American validation, but the ERC has not been validated in a Norwegian sample. The ERC consists of two subscales, the Emotion Regulation subscale (ER) and the Lability/Negativity subscale (L/N). The ER subscale measures appropriate emotional expression, empathy and emotional self-awareness; high scores reflect good emotion regulation. The L/N subscale measures inflexibility, lability and dysregulation. Higher scores reflect dysregulation. The mean item score was calculated individually for each subscale. In the present sample, Cronbach’s alphas were acceptable-to-good for maternal (ERC ER α = 0.72, ERC L/N α = 0.81) and paternal (ERC ER α = 0.79, ERC L/N α = 0.80) reports.

#### The Hopkin‘s symptom checklist (HSCL-10)

The HSCL-10 is a 10-item self-report questionnaire measuring adult symptoms of anxiety and depression within the previous week. Higher scores indicate higher levels of symptoms. The HSCL-10 is a short version of the HSCL [42]. The HSCL-10 has been validated with a Norwegian sample [43]. Cronbach’s alphas in our sample were good for both mothers’ (α = 0.87) and fathers’ (α = 0.85) reports.

#### Early adolescence stress questionnaire (EASQ)

The EASQ was originally based on several questionnaires regarding youth stressors, with additional items adjusted to children and adolescents in Norway. In the present study, the EASQ was reported by parents. The questionnaire contains 22 items describing stressors over the previous 12 months, covering areas regarding family, self, friends and school. Both acute negative life events and chronic stress are included [44]. The EASQ measures the cumulative load of unrelated stressors that the child have experienced, therefore reliability scores are uninformative. Example questions are “Has your child switched schools?” and “Has someone close to the child died?”. All answers are given as Yes or No, and all items contribute to the sum score.

### Statistics

Analyses were performed using IBM SPSS 23. We used paired t-tests to compare scores of symptoms and emotion regulation between respondents. Bivariate correlations between relevant variables were also tested.

Hierarchical multiple regressions were preformed to determine whether emotion regulation adds to the explained variance of the control variables on children’s symptom levels of anxiety or depression. All assumptions of linear regression were met, and levels of multicollinearity and homoscedasticity were acceptable. Step one in the hierarchical regression included all control variables, and step two also included the emotion regulation variables. The dependent variables were children’s symptom of anxiety and depression, as reported by children themselves, mothers and fathers. Paternal scores on the control variables of paternal education level, paternal mental health and the child’s experience of stressful life events were used in the regressions with paternal scores of children’s emotion regulation. In the regressions with maternal reports of emotion regulation scores, we used maternal reports of the same control variables. In addition, we conducted similar hierarchical regression analyses using reports from the opposite parent (e.g. measuring whether maternal report of emotion regulation would predict paternal report of childhood anxiety/depression or vice versa).

Of the 624 children with parents participating in the study, 22 ERC reports were missing, and therefore 602 cases were analyzed. Due to aspects of computerized data collection, no participants had any single items missing. In the regression analyses, missing values were excluded list-wise, resulting in the exclusion of four maternal and three paternal responses.

To compare the relationship between symptoms and emotional regulation for maternal and paternal results, we used the Paternoster test [45]. The Paternoster test is used to test if an empirical relationship estimated in two independent samples are similar, by comparing the unstandardized regressions coefficients from the two independent regressions.

## Results

Descriptive data are presented in Table 1. Compared to fathers, mothers scored their children higher on the ERC ER (*r* = 0.40, *CI* = [− 1.25, − 0.32], *p* < 0.001). For the ERC L/N, there were no significant differences between parental scores.

**Table 1:** Descriptive statistics split by respondents

	Child *n* = 602 (1)	Mother *n* = 537 (2)	Father *n* = 289 (3)	Groups (t-test)
	M (SD)	M (SD)	M (SD)	
Child age	10.07 (0.90)			
Child gender	Girls 58.10%			Girls>Boys***
MASC (0–117)	63.43 (13.78)	43.39 (15.37)	41.36 (14.67)	1 > 2,3***
SMFQ (0–26)	9.92 (4.91)	5.64 (4.86)	5.08 (4.58)	1 > 2,3***, 2 > 3**
ERC L/N (0–45)		11.26 (5.96)	11.37 (5.82)	n.s.
ERC ER (0–24)		18.99 (3.30)	18.33 (3.26)	2 > 3**
HSCL (0–30)		4.07 (4.36)	3.23 (3.71)	
EASQ (0–44)		1.60 (1.62)	1.43 (1.44)	
Economy (5 point scale. 1 = 350,000 NOK, 5 = over 1 million NOK)		3.71 (1.19)	3.71 (1.19)	
Education (5 point scale, 1 = ten years of primary school, 5 = four years or more on college/university)		3.93 (0.98)	3.81 (1.07)	

All scores are sum-scores. Economy is measured per family. ERC L/N high score indicates poor regulation skills. ERC ER high score indicates good regulation skills

*MASC* Multidimensional Anxiety Scale for Children, *SMFQ* Mood and Feeling Questionnaire – short form, *ERC* Emotion regulation checklist, *HSCL* The Hopkin‘s symptom check list, *EASQ* Early Adolescence Stress Questionnaire

*p = < 0.05. **p = < 0.01. ***p = < 0.001

The correlation between the symptoms score and emotion regulation ranged between 0.68 (*p* < 0.001) for depression and ERC L/N reported by fathers and 0.00 (*p* > 0.05) for child-reported anxiety scores and maternal scores on the ERC ER (Table 2).

**Table 2:** Correlation matrix

	MASC-C	MFQ-C	MASC-M	MFQ-M	ERC LN-M	ERC ER-M	MASC-F	MFQ-F	ERC L/N-F	ERC ER-F
MASC-C	1									
MFQ-C	0.32***	1								
MASC-M	0.24***	0.14***	1							
MFQ-M	0.10*	0.29***	0.56***	1						
ERC L/N-M	0.04	0.18***	0.42***	0.59***	1					
ERC ER-M	−0.00	− 0.13**	0.37***	− 0.53***	− 0.53***	1				
MASC-F	0.23***	0.06	0.56***	0.39***	0.27***	− 0.28***	1			
MFQ-F	0.10	0.34***	0.35***	0.61***	0.45***	−0.41***	0.53***	1		
ERC L/N-F	0.11	0.24***	0.32***	0.51***	0.57***	−0.38***	0.45***	0.68***	1	
ERC ER-F	−0.10	−0.21***	− 0.28***	−0.34***	− 0.34***	−0.40***	− 0.34***	−0.41***	− 0.51***	1

Children *n* = 602, Mother *n* = 537, Father *n* = 289

*MASC* Multidimensional Anxiety Scale for Children, *SMFQ* Mood and Feeling Questionnaire – short form, *ERC* Emotion regulation checklist. *C* Reported by child, *M* Reported by mother, *F* Reported by father

*p = < 0.05. **p = < 0.01. ***p = < 0.001

### Regression analyses

#### Anxiety symptoms

When the child’s self-report on MASC (anxiety) was the dependent variable, none of the ERC (emotion regulation) subscales contributed to the model; this was true for both maternal and paternal reports.

When the maternal report on MASC was the dependent variable, both ERC subscales contributed significantly to the model (L/N: *β* = 0.24, *p <* 0.001, ER: *β =* − 0.16, *p <* 0.001), *∆R*^*2*^ = 10.2% (Table 3). When the paternal report on MASC was the dependent variable, both ERC subscales contributed significantly to the model (L/N: *β* = 0.30, *p* < 0.001, ER: *β* = − 0.13, *p* < 0.05), *∆R*^*2*^ = 12.5% (Table 3). The Paternoster test was used to compare the unstandardized regression coefficients (b_1_) between regressions containing parental reports on MASC and ERC; there was no difference (L/N: *Z* = 0.6, *p* < 0.05, ER *Z* = 0.4, *p <* 0.05).

**Table 3:** Hierarchical multiple regression analysis, Anxiety (MASC)

Variables	Fathers’ reports on child anxiety symptoms as dependent, fathers’ reports on control and independent variables (*n* = 285)					Mothers’ reports on child anxiety symptoms as dependent, mothers’ reports on control and independent variables (*n* = 534)				
	β (CI)	t	Part^2^	Total R^2^	**∆** ***R*** ^***2***^	β (CI)	t	Part^2^	Total R^2^	**∆** ***R*** ^***2***^
Step 1				18.10%	**19.80%**				16.20%	**17.10%**
Age (child)	0.10 (−0.09, 3.55)	1.87				0.12 (0.73, 3.41)	3.04**			
Gender (child)	0.11 (0.16, 6.58)	2.07*				0.04 (− 1.34, 3.55)	0.89			
Economy (family)	−0.11 (− 3.01, − 0.10)	− 1.84				−0.09 (− 2.30, − 0.10)	− 2.14*			
Parental Education	0.00 (− 1.48, 1.60)	0.08				−0.00 (−1.34, 1.30)	− 0.03			
Stress (EASQ)	0.01 (−1.09, 1.28)	0.15				0.14 (0.54, 2.16)	3.29***			
Parental psychiatric health (HSCL)	0.40 (1.12, 2.03)	6.84***				0.30 (0.76, 1.34)	7.08***			
Step 2				30.40%	**12.50%**				26.20%	**10.20%**
Age (child)	0.09 (− 0.09, 3.26)	1.86				0.12 (0.71, 3.23)	3.07*			
Gender (child)	0.15 (1.40, 7.34)	2.90**				0.07 (−0.23, 4.39)	1.77			
Economy (family)	−0.12 (−3.08, − 0.21)	−2.25*				− 0.09 (−2.15, 0.09)	−2.14*			
Parental Education	0.04 (−0.91, 1.95)	0.72				0.03 (−0.74, 1.76)	0.80			
Stress (EASQ)	−0.05 (−1.57, 0.65)	− 0.82				0.07 (− 0.15, 1.40)	1.58			
Parental psychiatric health (HSCL)	0.26 (0.59, 1.48)	4.59***				0.19 (0.37, 0.94)	4.50***			
ERC Liability/Negativity (L/N)	0.30 (0.46, 1.08)	4.85***	5.76%			0.24 (0.39, 0.86)	5.26***	3.84%		
ERC Emotion regulation (ER)	−0.13 (−1.19, − 0.05)	−2.13*	1.10%			− 0.16 (−1.18, − 0.34)	−3.57***	1.77%		

All scores are sum-scores. ERC L/N high scores indicate poor regulation skills. ERC ER high scores indicate good regulation skills

*SMFQ* Mood and Feeling Questionnaire – short form, *ERC* Emotion regulation checklist, *HSCL* The Hopkin‘s symptom check list, *EASQ* Early Adolescence Stress Questionnaire

*p = < 0.05. **p = < 0.01. ***p = < 0.001

In addition, we tested whether paternal report of emotion regulation would predict maternal report of childhood anxiety or vice versa. Paternal report of children’s emotional regulation predicted maternal report of MASC only for the L/N subscale of ERC (L/N: *β =* 0.17, *p* < 0.05), ∆R^2^ = 5.60%, while maternal report of children’s emotional regulation predicted paternal report of MASC only for the ER subscale of ERC (ER: *β =* − 0.20, *p* < 0.01), ∆R^2^ = 7.20%.

#### Depressive symptoms

When the child’s self-report on SMFQ (depression) was the dependent variable and maternal reports were used as the independent variables, ERC L/N contributed significantly to the model (β = 0.12, *p* < 0.05), ∆R^2^ = 1.8% (Table 4). When the child’s report on SMFQ was the dependent variable and paternal reports were used on the independent variables, ERC did not contribute to the model.

**Table 4:** Hierarchical multiple regression analysis, Depression (SMFQ)

Children’s self-reports on child depression symptoms as dependent, mothers’ reports on control and independent variables (*n* = 534)					
Variables	β (CI)	t	Part^2^	Total R^2^	**∆** ***R*** ^***2***^
Step 1				4.50%	**5.60%**
Age (child)	0.12 (0.20, 1.10)	2.84**			
Gender (child)	0.08 (−0.09, 1.56)	1.76			
Economy (family)	0.05 (−0.16, 0.58)	1.14			
Maternal Education	−0.04 (− 0.64, 0.26)	−0.84			
Stress (EASQ)	0.16 (0.20, 0.75)	3.43***			
Maternal psychiatric health (HSCL)	0.06 (−0.03, 0.16)	1.34			
Step 2				6.00%	**1.80%**
Age (child)	0.12 (0.20, 1.09)	2.82**			
Gender (child)	0.09 (0.03, 1.68)	2.03*			
Economy (family)	0.06 (−0.14, 0.59)	1.20			
Maternal Education	−0.02 (− 0.56, 0.33)	−0.51			
Stress (EASQ)	0.13 (0.10, 0.65)	2.67**			
Maternal psychiatric health (HSCL)	0.01 (−0.09, 0.12)	0.31			
ERC Liability/Negativity (L/N)	0.12 (0.01, 0.18)	2.28*	0.92%		
ERC Emotion regulation (ER)	−0.05 (−0.22, 0.08)	−0.97	0.18%		

All scores are sum-score. ERC L/N high scores indicate poor regulation skills. ERC ER high scores indicate good regulation skills

*SMFQ* Mood and Feeling Questionnaire – short form, *ERC* Emotion regulation checklist, *HSCL* The Hopkin‘s symptom check list, *EASQ* Early Adolescence Stress Questionnaire

*p = < 0.05. **p = < 0.01. ***p = < 0.001

When the maternal report on SMFQ was the dependent variable, both ERC subscales contributed significantly to the model (L/N: *β* = 0.34, *p <* 0.001, ER: *β* = − 0.25, *p <* 0.001), *∆R*^*2*^ = 21.6% (Table 5). When the paternal report of SMFQ was the dependent variable, only the L/N subscale of ERC contributed significantly to the model (L/N: *β* = 0.53, *p <* 0.001), *∆R*^*2*^ = 28.0% (Table 5). The Paternoster test was used to compare the unstandardized regression coefficients (b_1_) between regressions containing parental reports on SMFQ and ERC. The ERC L/N paternal reports were higher than the maternal reports (*Z* = 2.8, *p <* 0.01). The ERC ER was only a predictor of children’s levels of depressive symptoms in maternal reports, and the Paternoster test was not calculated.

**Table 5:** Hierarchical multiple regression analysis, Depression (SMFQ)

Variables	Fathers’ reports on child depression symptoms as dependent, fathers’ reports on control and independent variables (*n* = 285)					Mothers’ reports on child depression symptoms as dependent, mothers’ reports on control and independent variables (*n* = 534)				
	β (CI)	t	Part^2^	Total R^2^	**∆** ***R*** ^***2***^	β (CI)	t	Part^2^	Total R^2^	**∆** ***R*** ^***2***^
Step 1				23.40%	**25.00%**				27.70%	**28.50%**
Age (child)	0.06 (−0.22, 0.88)	1.18				0.07 (−0.03, 0.76)	1.83			
Gender (child)	0.02 (−0.78, 1.16)	0.38				−0.06 (−1.34, 0.09)	−1.71			
Economy (family)	0.04 (−0.28, − 0.66)	0.80				−0.02 (− 0.40, 0.25)	−0.46			
Parental Education	0.07 (−0.77, 0.16)	−1.28				0.04 (−0.19, 0.59)	1.00			
Stress (EASQ)	0.20 (0.29, 1.00)	3.55***				0.30 (0.67, 1.14)	7.51***			
Parental psychiatric health (HSCL)	0.38 (0.34, 0.61)	6.81***				0.35 (0.31, 0.48)	9.01***			
Step 2				51.60%	**28.00%**				49.40%	**21.60%**
Age (child)	0.05 (−0.17, 0.70)	1.19				0.06 (−0.02, 0.64)	1.88			
Gender (child)	0.07 (−0.13, 1.42)	1.64				−0.02 (− 0.77, 0.44)	−0.53			
Economy (family)	0.02 (−0.29, 0.46)	0.46				−0.01 (− 0.31, 0.23)	−0.29			
Parental Education	−0.01 (− 0.42, 0.32)	−0.27				0.09 (0.11, 0.76)	2.62**			
Stress (EASQ)	0.12 (0.08, 0.66)	2.54*				0.19 (0.37, 0.78)	5.55***			
Parental psychiatric health (HSCL)	0.19 (0.12, 0.35)	4.01***				0.19 (0.14, 0.29)	5.53***			
ERC Liability/Negativity (L/N)	0.53 (0.34, 0.50)	10.20***	17.72%			0.34 (0.22, 0.34)	8.92***	7.56%		
ERC Emotion regulation (ER)	−0.08 (−0.27, 0.03)	−1.64	0.46%			−0.25 (− 0.48, − 0.26)	−6.68***	4.24%		

All scores are sum-score. ERC L/N high scores indicate poor regulation skills. ERC ER high scores indicate good regulation skills

*SMFQ* Mood and Feeling Questionnaire – short form, *ERC* Emotion regulation checklist, *HSCL* The Hopkin‘s symptom check list, *EASQ* Early Adolescence Stress Questionnaire

*p = < 0.05. **p = < 0.01. ***p = < 0.001

In addition, we tested whether paternal report of emotion regulation would predict maternal report of childhood depression or vice versa. Paternal report of children’s emotional regulation predicted maternal report of SMFQ only for the L/N subscale of ERC (L/N: *β =* 0.38, *p* < 0.001), ∆*R*^*2*^ = 14%, while maternal report of children’s emotional regulation predicted paternal report of SMFQ for both the L/N and ER subscales (L/N: *β =* 0.26, *p* < 0.001, ER: *β =* − 0.23, *p* < 0.01), ∆*R*^*2*^ = 21.9%.

## Discussion

The present study investigated emotion regulation in relation to anxious and depressive symptoms in children aged 8–12 years.

When parental reports of symptoms were used, the results supported our first hypothesis. We found a negative association between children’s symptoms of anxiety and depression and emotion regulation. These results were retained even after controlling for known risk factors such as parental mental health, SES, stress the preceding year, and the child’s age and gender. The results are in line with the work by Kovacs and Yaroslavsky [46], who found deficits in emotion regulation to be evident in children at risk for depression, and with Schneider and colleges [21] who found negative emotion regulation skills to be a risk factor for anxiety symptoms.

Our findings indicated that a lack of positive strategies to regulate emotions, as well as the presence of negative emotion regulation strategies, were associated with anxious and depressive symptoms. Such regulation strategies should therefore be explored in longitudinal studies as potential targets for intervention. Our results show the same tendency as the findings from the longitudinal study of Kim-Spoon and colleges [15], who found low positive emotion regulation and high dysregulation to be independent predictors of internalizing symptoms in children. By separating the measurement of anxiety and depression, the present study further elaborated these findings. The results from the present study are also supported by theories that underlying deficits in emotion regulation are a risk factor for depression and anxiety [12–14].

Our study is based on cross-sectional data, and therefore we cannot state the direction of the relationships [47]. Symptoms of anxiety and depression might weaken the child’s emotion regulation capacities, leading to repeated failure to downregulate negative feelings and upregulate positive feelings, thus weakening the child’s belief in their capability to influence their own feelings. Worsening of internalizing symptoms might also increase the intensity of emotions and thereby the child’s difficulties in regulating them [48]. There is not necessarily a contradiction between deficits in emotion regulation being a potential risk factor for the disorder and increased difficulties with emotion regulation over the course of the disorder. Transactional relationships between several factors working together in developing and maintaining disorders are a widely accepted theory within the field of child psychopathology [49].

Inclusion in the present study was based on elevated symptoms of anxiety and/or depression; thus, this was not a sample of clinically depressed or anxious children. The relationship between symptoms and poor emotion regulation in this sample supports the notion that deficits in emotion regulation are detectable in children with subclinical internalizing symptoms. Therefore, emotion regulation is a potentially important target in prevention and identification of children at risk.

However, based on the child’s report, our first hypothesis was only confirmed regarding depressive symptoms and maternal reports of emotion regulation. One possible interpretation of this could be that the association between internalizing symptoms and emotion regulation is not that strong, and other factors should be emphasized in transdiagnostic research and interventions. Still, studies have repeatedly found only medium agreement between children’s self-reports and caregivers’ reports, with no clear answer regarding whose reports are most accurate [50]. Both child and parental reporters provide clinically meaningful information, enlightening a phenomenon from different angles [25]. Caution must be taken, as the results did not show an association between emotion regulation and symptom scores from all the informants.

Our results only partially supported our second hypothesis: No difference was found between parental reports regarding the association of anxiety symptoms and emotion regulation in children. This might indicate that there is no difference between parental reports regarding this association. Another potential explanation is that our sample size of fathers was too small to detect differences.

The results show parental differences for the association between children’s emotion regulation and depressive symptoms. Children might display different emotion regulation behaviors to their parents, reflecting differences in parent-child relationships [25]. Parents might also have dissimilar interpretations and weightings of their children’s behavior [27]. Alternatively, mothers may more accurately see and report the positive emotion regulation behaviors of their children. Compared to fathers, mothers reported higher levels of the ER subscale of ERC, which captures positive emotion regulation behaviors in children. Still another possibility is that mothers idealize more and that paternal reports are more accurate.

In the additional analyses using opposite parental reporters of emotion regulation and of depressive and anxiety symptoms, the levels of symptoms were negatively associated with emotion regulation, though with a slightly altered regulation profile compared using same reporters. Paternal report of anxiety symptoms in children, was associated with maternal report of ER, while maternal report of anxiety in children was associated with paternal report of LN, both results confirm the findings from the main analyses. As for depression, maternal report of depressive symptoms was associated with paternal LN, and paternal reports with both the ERC scales as reported by the mother, also a similar pattern as in the main analyses.

These findings may indicate that fathers more accurately see and report the dysregulation (LN) of emotion regulation behaviors of their depressed and anxious children as reported by mothers. While mothers see and report lack of positive emotion regulation behaviors of their anxious children as reported by fathers. Mothers also see and report both lack of positive emotion regulation and dysregulation of their depressed children, independent of whether depressive symptoms is reported by mother or father. All over, the additional analyses with opposite reporters thus strengthen the results in the present study, especially regarding ERC and depressive symptoms.

The difference in association between emotion regulation and depression implies that both parents contribute important information in understanding their children’s difficulties. Combining maternal and paternal reports therefore holds the potential to broaden our understanding of the association between depressive symptoms and emotion regulation.

One explanation of differences in parental evaluations of their children’s mental status has been proposed to be linked to the parents’ own state of mind [51]. In the present study, however, we have controlled for parental psychological problems. The results might therefore give a correct picture of how parents differ in their conceptions of their children’s ability to regulate emotions in relation to depressive symptoms, in contrast to how parents differ with respect to anxiety symptoms.

Importantly, research including both paternal and maternal data often finds parental differences [25, 27]. Regardless of the explanation, it seems that in both research and clinical work with children at risk for internalizing problems, both caregivers should be included if possible [52]. Informant differences are interesting beyond the simple question of whether there are differences in reported symptoms: they are also interesting in understanding relationships between symptoms and constructs of emotion regulation.

### Strengths and limitations

This study used a large national sample of Norwegian children reporting elevated anxious and/or depressive symptoms. Few exclusion criteria ensured a diverse sample. Including fathers in the parental sample addresses an important gap in the research literature [25]. However as children in our study were recruited on the basis of their self-reported elevated anxious and/or depressive symptoms, further research will be required to test whether these findings generalize to the general population. Furthermore, the sample is skewed toward well educated parents, especially for mothers, indicating that our sample are skewed towards higher SES. As low SES are associated with increased risk for psychopathology symptoms in children [31], the skewness in our sample possibly reduce generalization of our results further.

The study should be repeated with emotion regulation measurements from both parent and child, as discrepancies between child and maternal reports of emotion regulation have been found [53]. Not having multiple informants allows the possibility that shared method variance could affect our results [54]. The relationship between emotion regulation and anxious symptoms was not statistical significant when children self-reported on anxious symptoms. As a result we cannot rule out that the association found for parental reports of anxious symptoms and emotion regulation was inflated by shared method variance. However, the relationship between emotion regulation and depressive symptoms was evident using only parental report for both measurements, and when children’s self-report on depressive symptoms was used as dependent variable. Although the effect diminished when different reporters were used, this may indicate that the relationship are not merely a result of measurement bias. However, whether parent or child reports are most accurate has not yet been clearly answered, and different informants might report on different aspects of the same construct [26]. Notably, Compas and colleges [3] compared studies using single and multiple informants on emotion regulation and found no moderator effect of the informant for the association between emotion regulation and internalizing symptoms.

This study was cross sectional. To establish emotion regulation as a possible risk factor for anxiety and depression, longitudinal data are necessary [47].

## Conclusion

Deficits in emotion regulation probably coexist with elevated symptoms of anxiety and/or depression in Norwegian children aged 8 to 12 years. Further, parental gender probably plays a differentiating role in the association between symptoms of depression and emotion regulation. This highlights the importance of including both parents in research and clinical work with children, as exclusion of one caregiver might bias our understanding of the child.

## Abbreviation

CBT: Cognitive behavioral therapy
EASQ: Early Adolescence Stress Questionnaire
ER: Emotion regulation, L/N lability/negativity
ERC: Emotional regulation scale
HSCL-10: Hopkins Symptom Checklist
IBM SPSS: International business machines statistical package for social sciences
MASC: Multidimensional Anxiety Scale for Children
NOK: Norwegian kroner
SD: Standard deviation
SES: Socioeconomic Status
SMFQ: Mood and feeling questionnaire – short version
